# Next-Generation Sequencing and Quantitative Proteomics of Hutchinson-Gilford progeria syndrome-derived cells point to a role of nucleotide metabolism in premature aging

**DOI:** 10.1371/journal.pone.0205878

**Published:** 2018-10-31

**Authors:** Jesús Mateos, Juan Fafián-Labora, Miriam Morente-López, Iván Lesende-Rodriguez, Lorenzo Monserrat, María A. Ódena, Eliandre de Oliveira, Javier de Toro, María C. Arufe

**Affiliations:** 1 Grupo de Terapia Celular y Medicina Regenerativa, Dpto. Ciencias Biomédicas, Medicina y Fisioterapia, Facultad de Ciencias de la Salud, Universidade da Coruña, INIBIC-CHUAC; 2 Cardiology Department, Health in Code, A Coruña, Spain; 3 Proteomics Platform–Barcelona Science Park, Barcelona, Spain; Southern Illinois University School of Medicine, UNITED STATES

## Abstract

Hutchinson-Gilford progeria syndrome (HGPS) is a very rare fatal disease characterized for accelerated aging. Although the causal agent, a point mutation in *LMNA* gene, was identified more than a decade ago, the molecular mechanisms underlying HGPS are still not fully understood and, currently, there is no cure for the patients, which die at a mean age of thirteen. With the aim of unraveling non-previously altered molecular pathways in the premature aging process, human cell lines from HGPS patients and from healthy parental controls were studied in parallel using Next-Generation Sequencing (RNAseq) and High-Resolution Quantitative Proteomics (iTRAQ) techniques. After selection of significant proteins and transcripts and crosschecking of the results a small set of protein/transcript pairs were chosen for validation. One of those proteins, ribose-phosphate pyrophosphokinase 1 (PRPS1), is essential for nucleotide synthesis. PRPS1 loss-of-function mutants present lower levels of purine. PRPS1 protein and transcript levels are detected as significantly decreased in HGPS cell lines vs. healthy parental controls. This modulation was orthogonally confirmed by targeted techniques in cell lines and also in an animal model of Progeria, the ZMPSTE24 knock-out mouse. In addition, functional experiments through supplementation with S-adenosyl-methionine (SAMe), a metabolite that is an alternative source of purine, were done. Results indicate that SAMe has a positive effect in the proliferative capacity and reduces senescence-associated Beta-galactosidase staining of the HPGS cell lines. Altogether, our data suggests that nucleotide and, specifically, purine-metabolism, are altered in premature aging, opening a new window for the therapeutic treatment of the disease.

## Introduction

Mutations in LMNA gene are the causal agent of subset of genetic diseases affecting mesoderm tissues called laminopathies [1]. This term refers to a highly heterogeneous group of disorders affecting the integrity of the nuclear lamina [2]. Among them, Hutchinson Guilford progeria syndrome (HGPS) or progeria is a fatal disease with a very low incidence characterized by a typical clinical picture of elderly pathologies [3]. HPGS-affected patients begin to show symptoms of accelerated aging at age of two, and usually die in the second decade of life due to cardiovascular deficiencies. HGPS is due, in most cases, to a point mutation (G608G) in LMNA gene encoding lamin A and C, major structural components of the nuclear lamina [4]. The mutation causes the occurrence of a cryptic alternative processing site in lamin A, generating a truncated isoform called progerin -PG- or Δ50 lamin A. This aberrant isoform remains farnesylated since the deletion includes the specific recognition sequence for the metalloprotease FACE1 that, in normal conditions removes the farnesylated C-terminal end of the Prelamin A to form the mature lamin A [5]. Accumulation of PG promotes defects on nuclear structure, replication, chromatin organization and stem cell differentiation, causing a senescence phenotype at the cellular level, and a premature aging of the organism [6,7]. A mouse model of HGPS, the knock-out deficiency for the metalloproteinase ZMPSTE24, the FACE1 homolog, fails to form mature lamin A, accumulates the permanently farnesylated precursor and recapitulates most of the symptoms of the disease [8].

So far, most of the efforts put on the reversion of the harmful effects of the nuclear accumulation of PG have focused on the use of farnesyl-transferase inhibitors (FTI) [9].

Aging is characterized by physiological alterations that compromise the health of the organism and represents one of the major risk factors for the development of a plethora of pathologies, including cardiovascular disease, cancer, musculoskeletal degeneration and neurodegenerative disorders [10]. At the molecular level, aging is accompanied by a set of hallmarks [11]. Among them, cellular senescence [12] mitochondrial dysfunction (13) [13], stem cell exhaustion [14], loss of proteostasis [15] and genomic instability [16] are the most characteristic. Aging is now considered a multi-factorial process. For this reason, the combination of complementary scientific approaches is necessary to fully understand the molecular aspects of aging and to delay or even reverse their detrimental aspects.

Proteomics provides tools to globally analyze cellular activity at protein level. Besides, this proteomic profiling will allow the elucidation of connections between broad cellular pathways and molecules that were previously impossible to predict using only traditional biochemical analysis. However, so far, the results obtained must be orthogonally validated with other approaches. Next-Generation Sequencing (NGS) technology, together with novel methods of pattern recognition and network analyses, has revolutionized cellular pathways [17]. Our intention for this study was to combine both shotgun proteomics and NGS to unravel new molecular pathways modulated in HGPS.

## Materials and methods

### Cell culture

Human HGPS-derived fibroblast cell lines (AG3513, AG3199 and AG8467) and their respective parental healthy controls (AG3512, AG3257 and AG8468) were acquired from Coriell Cell Repositories (CCR, Camdem, NJ, USA). Cells were cultured according to CCR instructions in Dulbecco’s modified Eagle’s medium (DMEM) supplemented with 10% fetal bovine serum (FBS) and 1% penicillin/streptomycin and expanded when they reached 70% confluence to 100mm culture dishes. For SAMe supplementation SAMe was periodically added to the medium at a concentration of 10 μg/mL.

### RNA isolation and preparation for NGS

Cells expanded in 100 mm culture dishes were harvested following standard procedures for RNA isolation using the TRIzol® extraction method. The quality of 1 μL of each RNA sample was checked using the Bioanalyzer 2100 (Agilent Techologies, St. Clara, USA) to determine the RIN (RNA Integrity) score using the 6000 Nanochip and reagents (Agilent). Samples with a RIN score > 7 were retained and converted to cDNA by SureSelect Strand Specific RNA library Prep for Illumina multiplexed sequencing method.

### Protein preparation for quantitative proteomics

Cells expanded in 100 mm culture dishes were gently scrapped with 200 μL of RIPA buffer with protease inhibitors at 4°C. Cells were transferred to 1.5 mL tubes and incubated at 100°C for 10 minutes. After centrifugation protein supernatants were collected for determination of the concentration by Bradford assay and checking of the protein integrity by SDS-PAGE and silver-staining.

### RNA sequencing

Sample preparation was carried out as recommended by Agilent SureSelect Strand- Specific RNA Library Prep (Agilent, St.Clara, United States) for Illumina multiplex sequencing method209. The study was designed to screen the complete transcriptome sequence per normal (AG03257 and AG03512) and HGPS (AG03199 and AG03513) human skin fibroblasts. RNA from AG08647 failed to be retro-transcribed and this cell line and its counter partner AG08648 were not included in the study. The experiment could not be repeated due to our tight budget and the lack of extra funding. 1 μg of total RNA per sample was used. Fragmented DNA was end-repaired and the sequencing data was generated on Hiseq 1500 (Illumina, San Diego, United States) on a rapid mode flow-cell (Illumina, San Diego, United States). All samples were sequenced twice and they were prepared in duplicate.

### Bioinformatics analysis of NGS data

An average of 25 million paired-end 100 bases pairs (bp) reads was obtained per sample in transcriptome analysis. The raw RNA-sequencing reads for each sample were aligned to the reference *Homo sapiens* (human) genome browser (GRCh38.p12) assembly using Bowtie2 (bowtie-bio.sourceforge.net/index.shtml/) and Tophat2 (http://tophat.cbcb.umd.edu/). After alignment, raw sequence read depths was converted to estimate transcript abundance measures as fragments per kilo base of exons per million (FPKM) values with Cufflinks (http://cufflinks.cbcb.umd.edu/). Identified transcripts were considered statistically significant when q-value ≤ 0.05.

### iTRAQ labeling

**Fig 1:**
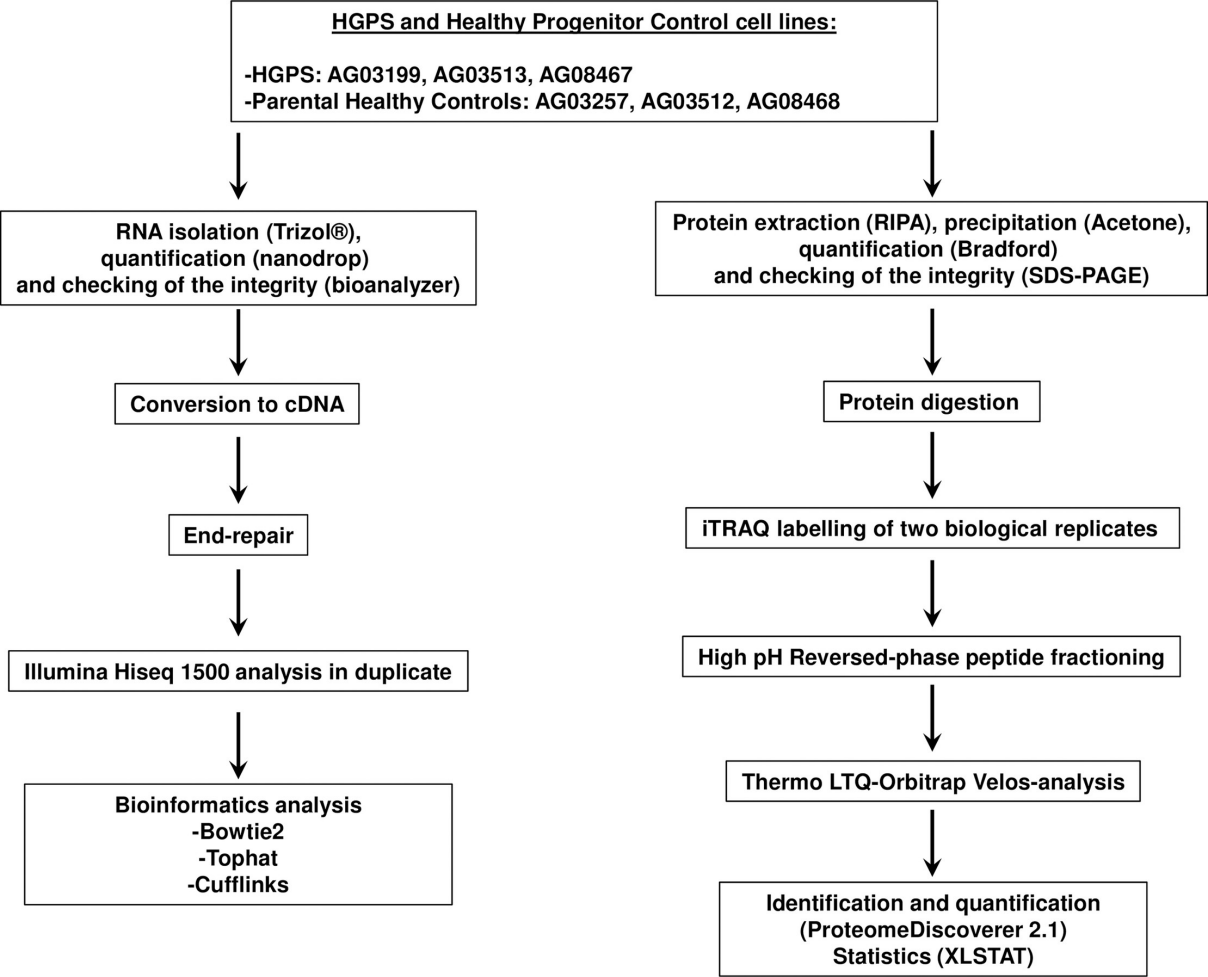
Parallel genomic and proteomic workflows. Schematic representation of the workflow followed for the genomic (left) and proteomic (right) large-scale approaches. HGPS and Healthy Progenitor cell lines were processed for RNA and total protein isolation. RNA was converted to cDNA for analysis in a Illumina Hi seq 1500 platform. Two technical replicates were analyzed for each sample. Raw data was processed and statistical analysis was done using Bowtie2, TopHat and Cufflinks programs. Total protein extracts were labeled using iTRAQ 8-plex. Two biological replicates were processed in parallel (iTRAQ1 and iTRAQ2). Labelled peptides were fractionated by Basic Reversed-phase chromatography and fractions were analyzed in a LTQ-Orbitrap Velos platform. Protein quantification was done using Proteome Discoverer 2.1 software and XLSTAT software was used for statistical analysis.

Total protein extracts (100 μg per condition) were processed for iTRAQ labeling following manufacturers instruction (Sciex). Two biological replicates were done in parallel (iTRAQ 1 and 2, Fig 1). Briefly, the total proteins were precipitated with overnight incubation with six volumes of cold acetone at −20°C, and denatured with 2% sodium dodecyl sulphate in 1 M triethylammonium bicarbonate buffer (ABSciex, Foster City, CA, USA). After incubation for 1 hour at 60°C using 50 mM tris-(2-carboxyethy) phosphine (Sciex), cysteines were blocked with 84 mM iodoacetamide (Sigma-Aldrich) at room temperature for 30 minutes in absence of ligth. Digestion was done with spectrometry-grade trypsin (Promega, Madison, WI, USA) at a concentration of 1:50 trypsin/protein for 16 hours at 37°C. The next morning each peptide solution was labeled for 1.5 hours at room temperature using the iTRAQ reagents (Sciex). Samples were labeled with iTRAQ reagents as follows: AG3513: tag 113; AG3199: tag 114; AG8467: tag 115; AG3512: tag 116; AG3257: tag 117; and AG8468: tag 118. As an internal control of the labeling a duplicate of AG3513 was labeled with tag 119. The reaction was stopped by reduction of the percentage of the organic phase with the addition of de-ionized water, and all the labeled samples were combined in one tube. Homemade stage tips were used for desalting prior LC analysis.

### High pH reversed phase fractionation

Each of the iTRAQ labeled tryptic digests was subjected to high pH fractionation with High pH reversed phase peptide fractionation kit (Pierce) following the manufacturer’s instructions. Briefly the samples were loaded onto a spin column in 0.1% trifluoroacetic acid (TFA), washed and buffer exchanged with high pH buffer and then eluted in 9 fractions of increasing acetonitrile (ACN) concentration (f1 = 10%ACN; f2 = 12.5%ACN; f3 = 15%ACN; f4 = 17.5%ACN; f5 = 20%ACN; f6 = 22.5%ACN; f7 = 25%ACN; f8 = 50%ACN; f9 = 75%ACN). Flow through and wash fractions were pooled and also analyzed as FT-wash fraction. The fractions were dried down in a speed-vacuum centrifuge and kept at -80°C until LC-MSMS analysis.

### LC-MSMS analysis

The 10 dried-down fractions were analyzed in a nano-Acquity liquid chromatograph (Waters) coupled to a LTQ-Orbitrap Velos (Thermo Scientific) mass spectrometer. Tryptic labeled peptides were resuspended in 2% ACN/1% formic acid (FA) solution and an aliquot (corresponding to 500 ng) was injected for their chromatographic separation. Peptides were trapped on a Symmetry C18^TM^ trap column (5μm 180μm x 20mm; Waters), and were separated using a C18 reverse phase capillary column (75 μm Øi, 25 cm, nano Acquity, 1.7μm BEH column; Waters). The gradient used for the elution of the peptides was 2 to 35% B in 155 minutes, followed by a gradient from 35% to 45% in 20 min (A: 0.1% FA; B: 100% ACN, 0.1%FA), with a 250 nL/min flow rate.

Eluted peptides were subjected to electrospray ionization in an emitter needle (PicoTip^TM^, New Objective) with an applied voltage of 2000V. Peptide masses (*m/z* 300–1800) were analyzed in data dependent mode where a full Scan MS in the Orbitrap with a resolution of 30,000 FWHM at 400m/z was obtained. Up to the 10 most abundant peptides (minimum intensity of 2000 counts) were selected from each MS scan and then fragmented using HCD (Higher Energy Collision Dissociation) in C-trap using nitrogen as collision gas, with 40% normalized collision energy and analyzed in the Orbitrap with a resolution of 7,500 FWHM at 400m/z. The scan time settings were: Full MS: 250 ms (1 micro-scan) and MSn: 300 ms (2 micro-scans).Generated .raw data files were collected with *Thermo Xcalibur (v*.*2*.*2)*.

Raw data was processed using Proteome Discoverer 2.1 software (Thermo Scientific) with SequestHT as search engine against the latest *SwissProt/Uniprot Human* database. 10 .raw files corresponding to the 10 injections from the MS analyses were used to perform as a single search against this database. Both a target and a decoy database were searched to obtain a false discovery rate (FDR; <1%), and thus estimate the number of incorrect peptide-spectrum matches that exceed a given threshold. The following search parameters were applied: Database/Taxonomy: SwissProt/Uniprot Human plus Contaminants (March 2017); Enzyme: Trypsin; Missed cleavage: 2; Fixed modifications: Carbamidomethyl of cystein, iTRAQ8plex (N-term), iTRAQ8plex (K); Variable modifications: oxidation of methionine, iTRAQ8plex (Y); Peptide tolerance: 10 ppm and 0.1 Da (respectively for MS and MS/MS spectra). Thermo software Proteome Discover (v.2.1) was also used for quantification, using an isobarically labeled reporter ion quantification method (iTRAQ 8-plex).

### Statistical analysis of iTRAQ data

XLSTAT software was used to analyze the individual ratios obtained after Proteome Discoverer 2.1 processing. Briefly, the ratios for all the proteins quantified in both replicates were extracted and classified as “equal” (twelve ratios HGPS/HGPS and Control/Control) or “opposite” (eighteen ratios Control/HGPS). After checking that the ratios follow a normal distribution using the Kolmogorov-Smirnov test we applied a Student T-test to identify those proteins that present significant (p-value ≤ 0.001) differences when comparing HGPS/HGPS and Control/Control (equal) *versus* Control/HGPS (opposite) ratios.

### Real-time PCR of PRPS1

RNA was transformed to complementary DNA (cDNA) using NZY First-Strand cDNA synthesis kit (NZYTECH, Lisbon, Portugal) according to manufacturer's instructions. cDNA was amplified using specific primers for the PRPS1 gene. The design of primers was carried out using the software Primer3 (http://biotools.umassmed.edu/bioapps/primer3). The sequences of the primers were as follows; Forward: tcagactgcctgctgacttc; Reverse: tacctcaacgtgctcagtgg. Quantitative RT-PCR was carried out in a LightCycler 480 Instrument (Roche Applied Science) using Light Cycler 4800 SYBR Green I Master kit (Roche, Basel, Switzerland). The amplification program consisted on initial denaturation of 92 ^o^C for 2 min followed by 40 cycles at 92 ^o^C for 15 s, annealing at 55–62 ^o^C, depending on the gene, for 30 s and extension at 72 ^o^C for 15 s. qRT-PCR were done in triplicate, with each set of assays repeated three times. For control experiments no reverse transcriptase was used.

### Immunohistochemistry of PRPS1 in liver sections

Full-depth sections (4 μm) of paraffin-embedded livers from ZMPSTE24 null, heterozygous and wild-type mouse strains (blocks kindly donated by B. Caramés, INIBIC, A Coruña, Spain) were cut with a microtome and fixed in 4% (w/v) paraformaldehyde (Sigma-Aldrich) in PBS at pH 7.6. All sections were immuno-stained with rabbit polyclonal antibody for PRPS1 (1:100) (Abcam). Biotinylated secondary antibodies were detected using a peroxidase-labeled biotin-streptavidin complex with diaminobenzidine substrate Ennvision Kit (Dako) following manufacturer instructions.

### Cell proliferation assay

Cell lines were cultured at a density of 10.000 cells/well in 96-well plates, with or without periodic addition of SAMe (Sigma-Aldrich) at a final concentration of 10 μg/mL. At different times of culture (3, 5, 7 and 10 days), 10 μl of Thiazolyl Blue Tetrazolium Bromide (MTT, from Sigma-Aldrich) was added per well to achieve a final concentration of 0.45 mg/ml and incubated by 4 hours at 37°C. The medium was removed and 100 μl of Dimethyl sulfoxide (Sigma-Aldrich) was added per well to dissolve formazan crystals for 10 min and absorbance was recorded at 570 nm in a Nanoquant platform (Tecam).

### Senescence-associated Beta-galactosidase staining

X-Gal cytochemical staining for SA-β-gal was performed as previously described [18]. The cells were fixed and then stained with freshly prepare SA-β-gal staining solution overnight at 37°C according to the manufacturer's protocols (Cell Signaling Technology, Beverly, MA, USA). The number of SA-β-gal-positive cells in randomly selected fields was expressed as a percentage of all cells counted.

## Results

### RNAseq analysis of HGPS and healthy control cell lines

**Fig 2:**
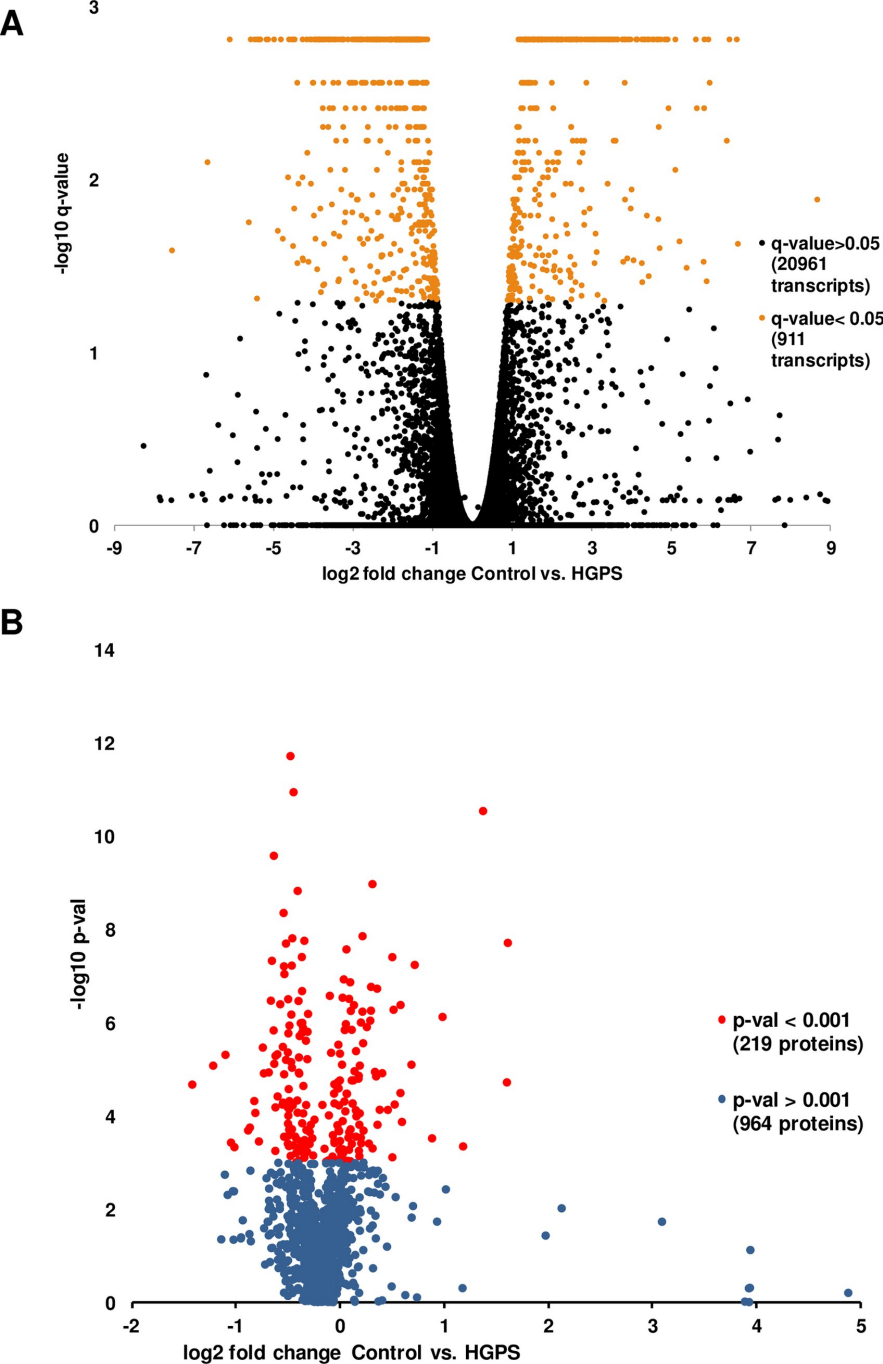
Transcripts and proteins significantly modulated in HGPS-derived cells versus healthy parental control cell lines. Volcano plot representations of the RNAseq (A) and shotgun proteomics (B) analysis of the HGPS and Control cell lines. For RNAseq, a threshold of q-value = 0.05 was set for significance according the statistical package used for analysis. A total of 911 transcripts present a q-value ≤ 0.05. For shotgun proteomics a threshold of p-value = 0.001 was set for significance according to the statistical package used. In this case 219 proteins were detected as significant with a p-value ≤ 0.001.

A schematic workflow of the analysis is represented in Fig 1. Genomic datasets are deposited at the Gene Expression Omnibus (GEO-NCBI) repository (http://www.ncbi.nlm.nih.gov/geo/). Raw files are public and freely accessible (www.ncbi.nlm.nih.gov/geo/query/acc.cgi?acc=GSE113648). A total of 21872 transcripts were detected and quantified in both conditions (Control and HGPS) by NGS following our approach (S1 File and Fig 2A) Among them, 911 were differentially expressed with a q-value ≤ 0.05 representing roughly a 4 per cent of the total sequenced transcriptome.

### iTRAQ analysis of HGPS and healthy control cell lines

Proteomic datasets are deposited at MassIVE repository (www.massive.ucsd.edu). Raw and processed files (Quantitative Proteomics Study (iTRAQ) of Progeria Cell Lines; #MSV000081576) are public and freely accessible. A summary of the quantitative proteomics analysis is represented in Fig 2B and S1 Fig, which show the quantification results in both experiments (S1A Fig), showing a nice correlation between the results (S1B Fig). Statistical analysis of the quantification results (S2 File and Fig 2B) showed that 219 proteins present significant (p-value ≤ 0.001) differences in the Control/HGPS *versus* Control/Control and HGPS/HGPS abundance ratios.

### Comparison of the proteomics and transcriptomics results

**Fig 3:**
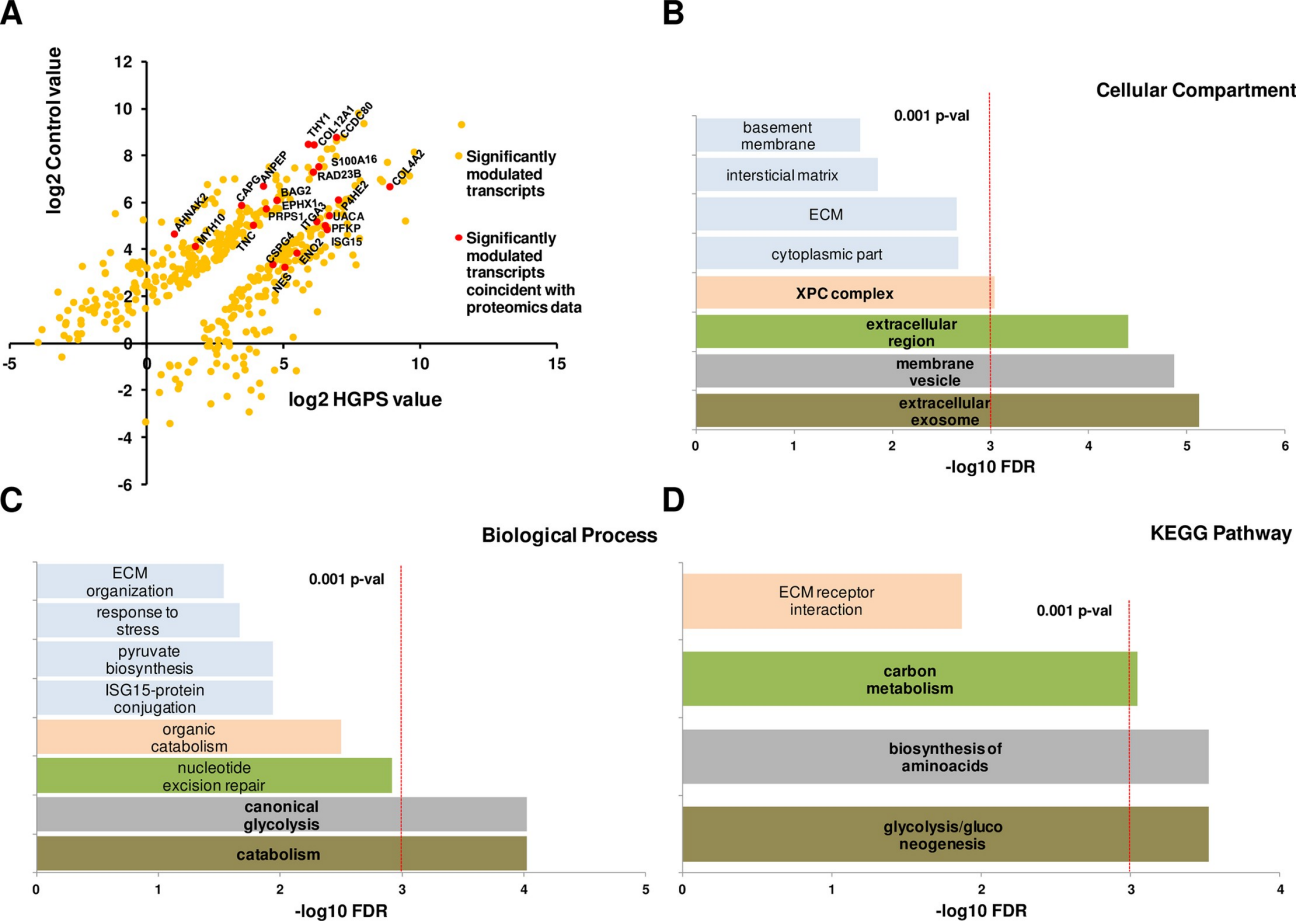
Crosschecking of the genomic and proteomic results shows that HGPS present features of a metabolic disorder. Representation of the modulation in HGPS *versus* Control cell lines of the transcripts detected as significant and those correlating with the proteomic approach (red dots in A). String 10.4 gene ontology statistical study of the 22 transcript/protein coincident pairs (B, C, D) demonstrate that most of the proteins are membrane-bounded or secreted and have a role on glycolysis, energy generation and synthesis of bio-molecules.

The difference in the amount of identified transcripts and proteins is inherent to the differences in the sensitivity of the techniques used. The list of significant proteins was crosschecked against the list of significant transcripts (Fig 3). From the 219 proteins we found that 24 of their corresponding transcripts were detected as differentially expressed with a q-value ≤ 0.05. As showed in Table 1, the correlation of those 24 protein/transcript pairs is very high. Most of those proteins appear to play important roles in cell shape and adhesion (THY1, TNC and COL12A1), proliferation and apoptosis (CSPG4) and metabolism and generation of energy (ENO2, PFKP, ANPEP and PRPS1). PRPS1 codifies for Ribose-phosphate pyrophosphokinase 1, a key protein responsible for *de novo* purine synthesis in the nucleotide metabolism. Our data showed a decrease of the amount of PRPS1 in HGPS cell lines at both transcript and protein levels when compared to the parental healthy controls. Due to the novelty of this link with HGPS, and the importance of de-regulation of the metabolism and imbalance of energy generation in this disease, we decided to focus our validation in this protein.

**Table 1:** Crosschecking of the RNAsec and ITRAQ data.

Gene name	Protein name	RNAsec data: Up in	iTRAQ data: Up in	Function
AHNAK2	Protein AHNAK2	Control	Control	Regulation of calcium channels.
ANPEP	Aminopeptidase N	Control	Control	Digestion of proteins.
BAG2	BAG family molecular chaperone regulator 2	Control	Control	Protein folding.
CAPG	Macrophage-capping protein	Control	Control	Cytoplasmic and nuclear structure.
CCDC80	Coiled-coil domain-containing protein 80	Control	Control	Extracellular matrix organization.
COL12A1	Collagen alpha-1(XII) chain	Control	Control	Extracellular matrix organization.
COL4A2	Collagen alpha-2(IV) chain	HGPS	HGPS	Extracellular matrix organization.
CSPG4	Chondroitin sulfate proteoglycan 4	HGPS	HGPS	Regulation of cell proliferation and migration
ENO2	Gamma-enolase	HGPS	HGPS	Glycolysis.
EPHX1	Epoxide hydrolase 1	Control	Control	Catabolism of aromatic compounds.
GPNMB	Transmembrane glycoprotein NMB	HGPS	Control	Cell migration and adhesion.
ISG15	Ubiquitin-like protein ISG15	HGPS	HGPS	Defense against bacteria.
ITGA3	Integrin alpha-3	HGPS	HGPS	Cell adhesion and cell-cell interaction.
MYH10	Myosin-10	Control	Control	Cell shape and cytokynesis.
NES	Nestin	HGPS	HGPS	Intermediate fillament binding.
P4HA2	Prolyl 4-hydroxylase subunit alpha-2	Control	HGPS	Proline hydroxilation in collagen.
PFKP	ATP-dependent 6-phosphofructokinase	HGPS	HGPS	Glycolysis.
PRPS1	Ribose-phosphate pyrophosphokinase 1	Control	Control	Nucleotide biosynthesis.
PSME2	Proteasome activator complex subunit 2	HGPS	HGPS	Proteosomal degradation.
RAD23B	UV excision repair protein RAD23 homolog B	Control	Control	Proteosomal degradation, part of the XPC complex.
S100A16	Protein S100-A16	Control	Control	Calcium binding protein, adipocyte differentiation.
THY1	Thy-1 membrane glycoprotein	Control	Control	Cell adhesion and cell-cell interaction.
TNC	Tenascin	Control	Control	Extracellular matrix organization.
UACA	Uveal autoantigen with coiled-coil domains and ankyrin repeats	HGPS	HGPS	Regulation of stress-induced apoptosis.

List of the 24 protein/transcript pairs selected after the crosschecking of the proteomics and transcriptomics data.

### Orthogonal validation of PRPS1 levels in HGPS and healthy control cell lines and in the ZMPSTE 24 KO mouse

**Fig 4:**
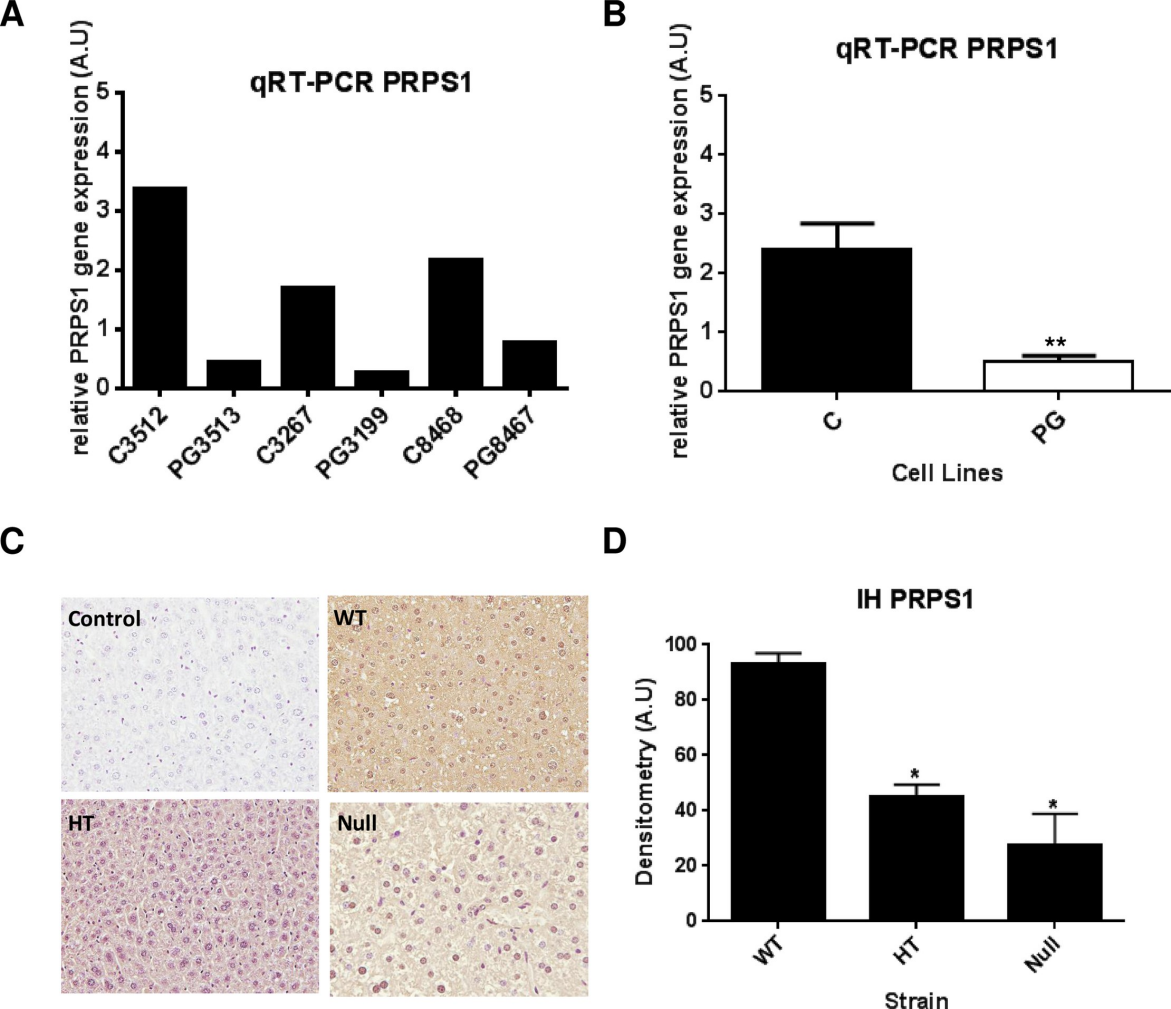
Targeted validation of PRPS1 shows down-regulation of transcript and protein levels in HGPS-cells and the animal model of HPGS, respectively. Orthogonal validation of PRPS1 modulation by targeted techniques in cell lines (A, B) and in the mouse model of HGPS (C, D). Real-Time PCR (RT-PCR) of HGPS and control cell lines (A) showing that PRPS1 transcript is down-regulated in the three HGPS cell lines when compare to their respective controls. Statistical analysis (B) demonstrates that the difference between control and HGPS is significant (p-value ≤ 0.01). Representative images (C) of the immunohystochemistry of the PRPS1 protein in liver sections of the ZMPSTE24-null mice strain (magnification: 20x). Statistical analysis shows that wild-type mice (WT) present significant (p-value ≤ 0.05) higher levels of the protein than heterozygous and null mice.

Quantitative Real Time PCR (qt-RT-PCR) analysis of PRPS1 in the cell lines used for the omics-study confirms the reduced levels of PRPS1 transcripts in the three HGPS cell lines (Fig 4A and 4B). Statistical analysis showed a significant difference (Fig 4B). We then decided to verify if this difference in expression occurs also *in vivo*. ZMPSTE24 knock-out mouse is the most used mammal model of HGPS. Since the liver is the main organ where purine *de novo* synthesis occurs, we checked PRPS1 levels by immunohistochemistry in liver sections of ZMPSTE24-null mice comparing to the heterozygous (HT) and the wild-type (WT) mice (Fig 4C). PRPS1 levels are significantly higher in WT *versus* both HT and null mice, thus corroborating the *in vitro* results.

### Study of the effect of the incubation with an alternative purine source in the proliferation capacity and senescence phenotype in HGPS cell lines

**Fig 5:**
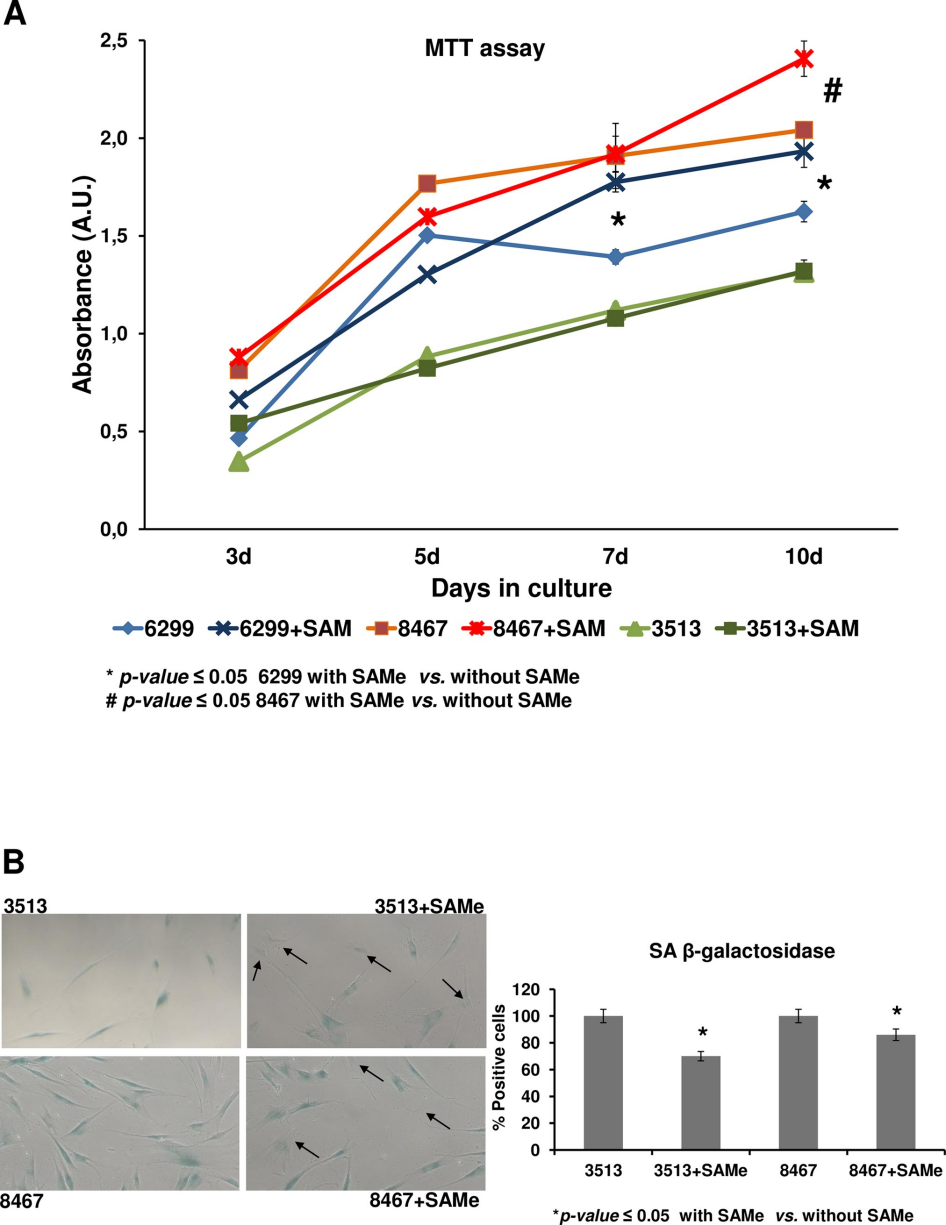
An alternative source of purine partially reverts the premature phenotype aging in HGPS-derived cells. Effect of incubation with SAMe on the premature-aging phenotype of HGPS cell lines. MTT-based proliferation assay (A) showing that periodic addition of SAMe to the culture media at a final concentration of 10 μg/mL, have a significant (p-value≤ 0.05) positive effect in the proliferation capacity of two out of the three cell lines after 10 days in culture. Representative images (magnification: 20x) of the staining of Senescence Associated Beta-galactosidase (SA-β-gal) staining in HGPS cells with and without SAMe addition to the culture media (B), showing a higher number of negative cells (arrows) in SAMe-treated cells. Densitometry analysis of the staining signal shows a decrease in two of the HGPS cell lines after incubation with SAMe (C).

To further investigate the link between cellular senescence in premature aging and defective purine synthesis we incubate HGPS cell lines with S-adenosyl methionine (SAMe), a compound that provides an alternative source of adenosine, the final metabolite in the purine synthesis pathway. The periodic addition of SAMe to the culture medium promotes a significant increase in the proliferative capacity of two out of the three HGPS cell lines studied (Fig 5A). This effect is accompanied by a decrease of senescence-associated β-galactosidase staining (Fig 5B).

## Discussion

Aging is a complex and multi-factorial process that, to the date, is poorly understood at the mechanistic level [19]. Genetic, epigenetic, metabolic and environmental factors contribute to the accumulation of molecular alterations that promote a negative imbalance in the regeneration/degeneration ratio of the tissues increasing the risk of disease and death at the organism level. Metabolism is a key factor influencing aging, since caloric restriction and down-regulation of the insulin/insulin-like growth factor 1 (IGF-1) pathway drive to an extension of the lifespan in nematodes, insects and mammals [20–22]. Premature aging syndromes share many molecular and phenotype features with aging-associated diseases [10]. The implication of de-regulation of the metabolism in accelerated aging has been also demonstrated. HGPS is characterized by a dramatic lipodystrophy [23] and has been recently associated to increased protein synthesis [15, 24].

Large-scale omic-techniques have experienced an exponential development in the last 5 years in terms of sensitivity, reproducibility and throughput capacity. These tools are now of great help to deeply investigate the molecular mechanisms underlying cell homeostasis and organism healthiness but also the altered molecular pathways in disease [25].

In the present work we have found a good correlation between the shotgun quantitative proteomics and the Next-Generation Sequencing after crosschecking of the results. The corresponding transcripts of 24 out of the 219 modulated proteins were detected as significantly modulated (Fig 3). As Table 1 shows, 22 out of the 24 protein/transcript pairs present the same kind of modulation, (i.e. increased in control or, alternatively, in HGPS in both approaches). Our intention for this study was to focus on this group of candidates. The analysis of the functional protein association networks using String 10.5 demonstrates that the crosschecked modulated proteins are mainly membrane-bounded or secreted and participate in glycolysis and metabolism (Fig 3), suggesting that HGPS presents features typical of a metabolic disorder.

Among them, one protein took specially our attention due to the novelty of its link with premature aging. PRPS1 gene codifies for Phosphoribosyl pyrophosphate synthetase-1, a key enzyme in nucleotide metabolism [26]. PRPS1 catalyzes the first step of the *de novo* Purine synthesis pathway, the formation of phosphoribosyl-pyrophosphate (PRPP) from Ribose-5-phosphate plus a molecule of adenosine triphosphate (ATP). PRPS1 loss-of-function mutations have been previously related to disease phenotypes like Arts Syndrome, diabetes insipidus or hearing loss [27].

*De novo* nucleotide synthesis is tightly regulated in the cells by a multi-enzymatic complex, the purinosome, integrated by six different enzymes that catalyze ten different reactions [28]. The resulting metabolites are essential for key biological processes such as cell signaling, energy generation and enzymatic activity, besides being the structural constituents of DNA and RNA [29]. The purinosome is a transient structure, that is subjected to periodic assembly/disassembly cycles in proliferating cells [30]. Purinosome activity is up-regulated during G1/S-phases, accompanied by an increase in PRPP intracellular levels, and decreases dramatically by G2/M-phases [31].

In the present study, PRPS1 levels are detected as significantly lower in HGPS vs. healthy progenitor cell lines in both massive approaches. This is, to our knowledge, the first scientific evidence linking premature aging and *de novo* purine synthesis. Further orthogonal verification by targeted techniques is in accordance with the results. Real-Time PCR demonstrated diminished levels of PRPS1 in the three HGPS cell lines *versus* the correspondent control cell lines. Then we decided to investigate if this down-regulation is observed *in vivo*. ZMPSTE24-null mouse is the most used animal model for the study of HGPS [32]. ZMPSTE24 is the mouse-homolog of human FACE1, the metalloprotease that converts pre-lamin A to mature lamin A. These mice accumulate permanently-farnesylated pre-lamin A, which exerts similar harmful effects than progerin, and display most of the phenotypic features of the disease [33]. Immunohistochemical analysis in ZMPSTE24-null mice liver sections showed diminished levels of PRPS1 when compared to both heterozygous and wild-type strains.

S-adenosyl-methionine (SAMe) is the central methyl donor in many cellular methylation reactions, catalyzed by cellular methyltransferases and hydrolases, and is also an alternative source of purine in living cells [34]. This metabolite was first discovered more than sixty years ago [35] and several studies have demonstrated its therapeutic value for the treatment of osteoarthritis [36], depression [37] and liver disease [38]. *In vitro*, SAMe has a positive effect on chondrocyte proliferation at a dose of 10 μg/mL [39]. In our hands, incubation of HGPS derived cells with SAMe ameliorates the senescence phenotype and increase the proliferation rate of the cells, suggesting that purine deprivation could be in part responsible for premature aging phenotype.

**Fig 6:**
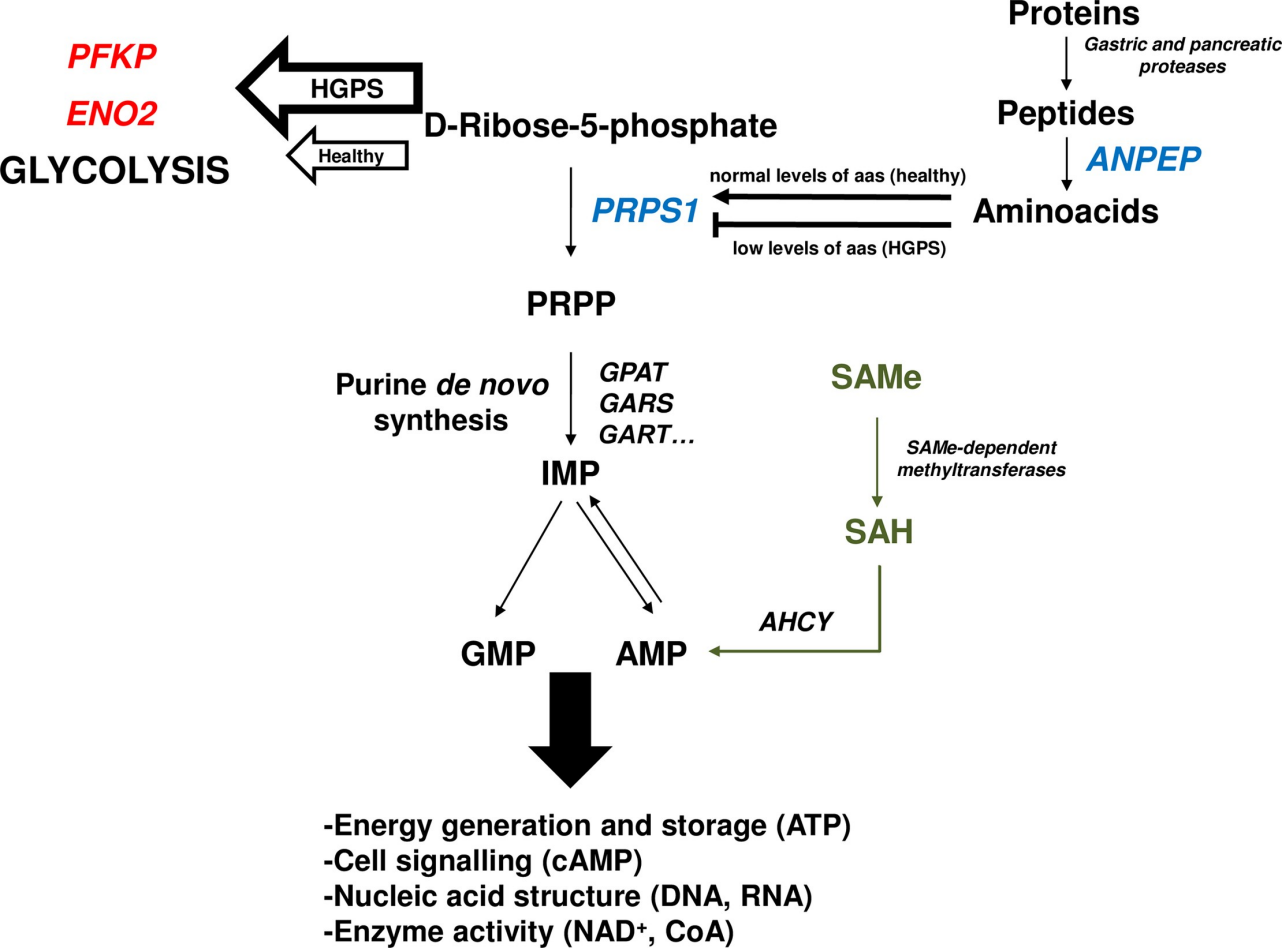
Proposed model to explain the down-regulation of PRPS1 in HGPS. Enzymes in blue are detected as down-regulated and those in red as up-regulated in the present study. High glycolitic rate in HGPS drives to a decrease in the levels of D-Ribose-5-phosphate available for *de novo* purine synthesis. This reduction compromises PRPS1 activity. Furthermore, this inhibition is complemented by free amino-acid starvation resulting from down-regulation of ANPEP. As a result, de novo nucleotide synthesis is affected thus promoting defects in key biological processes that contribute to the premature aging phenotype. Supplementation of SAMe helps to restore the levels of AMP which, in turn, could be transformed to GMP by the cellular machinery, partially ameliorating the premature aging phenotype. Abbreviations: PFKP: ATP-dependent 6-phosphofructokinase; ENO2: gamma-enolase; PRPS1: ribose-phosphate pyrophosphokinase 1; ANPEP: aminopeptidase N; GPAT: glutamine-phosphorybosil aminotransferase; GARS: glycinamide-ribonucleotide synthetase; GART: glycinamide-ribonucleotide transformylase; IMP: inosine monophosphate; GMP: guanosine monophosphate; AMP: adenosine monophosphate; SAMe: S-adenosylmethionine; SAH: S-adenosylhomocysteine; AHCY: S-adenosylhomocysteine hydrolase.

PRPS1 activity has been previously reported to be negatively modulated by both amino-acid starvation and a decrease of available D-Ribose-5-phosphate [40]. D-Ribose-5-phosphate is an intermediate of the pentose phosphate pathway, specifically the first product of the non-oxidative set of reactions. Interestingly, glycolysis has been also described as enhanced in ZMPSTE24-null mice using proteomics approaches [41]. This is consistent with our results, since we have found in the present work two of the key glycolytic enzymes, ATP-dependent 6-phosphofructokinase (PFKP) and gamma-enolase (ENO2), as up-regulated in HGPS cell lines. In this scenario (Fig 6), the increase in the glycolysis due to lamin A de-regulation, would produce a decrease in the amount of available D-Ribose-5-phosphate necessary for purine biosynthesis, thus promoting a reduction in the activity of PRPS1 affecting the entire *de novo* purine pathway. On the other hand, regarding the effect of amino-acid starvation on the PRPS1 activity, it is noteworthy the reduction of the levels of aminopeptidase N (ANPEP) that we have detected by both large-scale approaches. ANPEP, also known as CD13, is one of the enzymes that participate in the final digestion of peptides coming from the gastric hydrolysis of proteins [42] and participates in the rennin-angiotensin system that regulates vasopressin release, thus regulating arterial blood pressure and the pathogenesis of hypertension [43]. A reduction of the levels of ANPEP in HGPS would be responsible for the inhibition of PRPS1 through amino-acid starvation and, even more importantly, would contribute to the hypertension and high blood pressure levels that, long term, derive in the cardiovascular malfunction characteristic of the disease.

Altogether, our results indicate a non-previously described alteration of the *de novo* purine synthesis cellular machinery in HGPS. An attractive possibility is that LMNA de-regulation promotes down-regulation of the PRPS1 gene, resulting in a decrease of PRPS1 levels in HGPS. In this case, purine synthesis deprivation would compromise energy generation and cell signaling. Further investigation is needed for a deeper understanding of this novel link between premature aging and de-regulation of purine synthesis and to explore new therapeutic windows for the treatment of the disease.

## Supporting information

S1 Fig Summary of the proteomic analysis. 749 proteins in common were quantificated in both iTRAQ replicates (A). Graphical representation of the Control/HGPS ratios shows a good correlation between the two replicates (B).
(TIF)

S1 File RNAseq analysis. RNAseq analysis of Control vs. HGPS cell lines.
(XLSX)

S2 File iTRAQ analysis. iTRAQ analysis (replicates 1 and 2) of Control cells vs. HGPS cell lines.
(XLSX)

## Abbreviations

HGPS: Hutchinson-Gilford progeria syndrome
PG: Progerin
NGS: Next-Generation Sequencing
iTRAQ: isobaric tags for absolute and relative quantitation
RIN: RNA integrity
RIPA: radioimmunoprecipitation assay
FDR: false discovery rate
PBS: phosphate buffered saline
qt-RT-PCR: quantitative Real-Time Polimerase Chain Reaction
PFKP: ATP-dependent 6-phosphofructokinase
ENO2: gamma-enolase
PRPS1: ribose-phosphate pyrophosphokinase 1
ANPEP: aminopeptidase N
GPAT: glutamine-phosphorybosil aminotransferase
GARS: glycinamide-ribonucleotide synthetase
GART: glycinamide-ribonucleotide transformylase
IMP: inosine monophosphate
GMP: guanosine monophosphate
AMP: adenosine monophosphate
SAMe: S-adenosylmethionine
SAH: S-adenosylhomocysteine
AHCY: S-adenosylhomocysteine hydrolase
FTI: farnesyltransferase inhibitors
