# Supplementary figures and images for: Next-Generation Sequencing and Quantitative Proteomics of Hutchinson-Gilford progeria syndrome-derived cells point to a role of nucleotide metabolism in premature aging
Source: PLoS One. 2018 Oct 31;13(10):e0205878. doi: 10.1371/journal.pone.0205878 (PMC6209416; doi:10.1371/journal.pone.0205878)

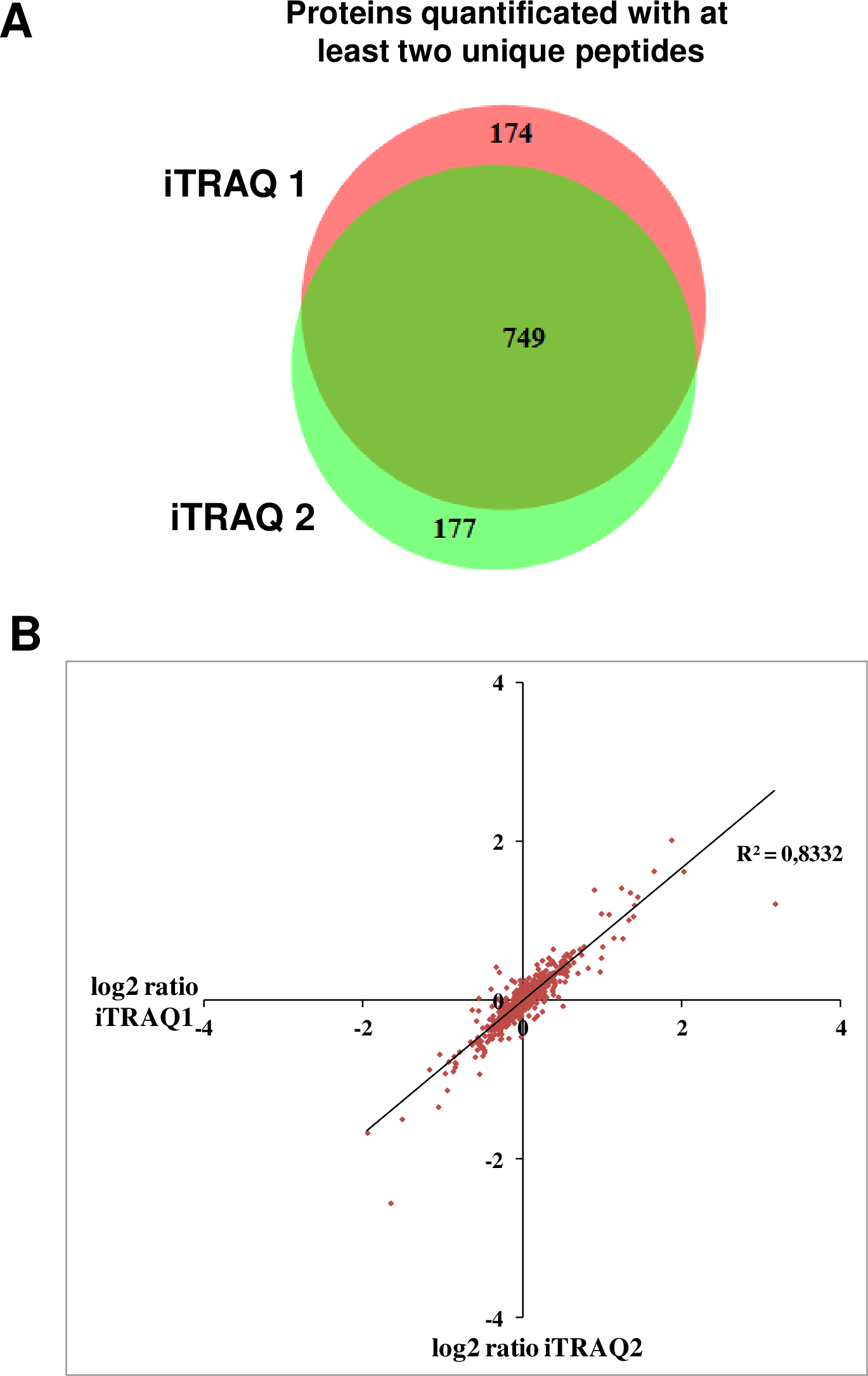

Supplement: S1 Fig — 749 proteins in common were quantificated in both iTRAQ replicates (A). Graphical representation of the Control/HGPS ratios shows a good correlation between the two replicates (B). (TIF) [file pone.0205878.s001.tif]
